# Emerging Strategies in Treating Corneal Alkali Burns: A Narrative Review

**DOI:** 10.7759/cureus.47662

**Published:** 2023-10-25

**Authors:** Mehul Mittal, Pravin Tidake, Mayank Kumar

**Affiliations:** 1 Ophthalmology, Jawaharlal Nehru Medical College, Datta Meghe Institute of Higher Education and Research, Wardha, IND; 2 Community Medicine, Jawaharlal Nehru Medical College, Datta Meghe Institute of Higher Education and Research, Wardha, IND

**Keywords:** crosslinked fibers, tulang honey, nanowafers, hyaluronic acid, alkali, cornea

## Abstract

Corneal alkali burns represent a complex and debilitating ocular injury, necessitating innovative strategies for effective management. This narrative medical review seeks to provide a comprehensive exploration of emerging approaches in the treatment of corneal alkali burns. The primary objectives of this review are multifaceted. First, we aim to unravel the intricate pathophysiology of corneal alkali burns, delving into the immediate and long-term consequences of alkali exposure on ocular tissues. Understanding the underlying mechanisms, including oxidative stress, inflammation, and neovascularization, is essential for the development of targeted therapeutic interventions. Second, we assess the efficacy of novel treatment modalities, encompassing pharmacological agents and surgical techniques, with a focus on their ability to mitigate corneal damage, facilitate tissue regeneration, and preserve visual function. By analyzing the latest clinical findings, we aim to identify promising avenues for improved patient outcomes.

Temporal dynamics play a crucial role in the healing process, and thus our review investigates the progression of corneal lymphangiogenesis and the expression patterns of key growth factors, such as vascular endothelial growth factor-C (VEGF-C). These insights into the timing of corneal healing provide valuable guidance for tailoring therapeutic interventions to specific stages of injury. Finally, we delve into regenerative therapies, particularly the potential of mesenchymal stem cells (MSCs) and their secretome as anti-inflammatory and antiangiogenic agents. By summarizing the promising results from preclinical and clinical studies, we illuminate the prospects of regenerative approaches in corneal alkali burn management. This narrative review aspires to serve as an indispensable resource for clinicians, researchers, and healthcare professionals involved in the treatment of corneal alkali burns. By addressing these objectives, we aim to foster a deeper understanding of this challenging condition, facilitate the development of innovative strategies, and ultimately enhance patient outcomes in the realm of corneal health and vision preservation.

## Introduction and background

Chemical damage to the cornea poses a significant risk to ocular health and visual function, with alkali exposure, even at minimal levels, leading to notable changes in corneal moisture content and light absorption [1]. Current clinical practice primarily relies on the frequent application of topical ophthalmic solutions for treating eye injuries, despite challenges in maintaining effective drug concentrations on the ocular surface [2]. To address these challenges, dexamethasone nanowafers (Dex-NWs) have been developed to gradually release medication [3].

This review highlights the results of investigations into Dex-NW administration, which have shown improvements in corneal clarity, reduced production of inflammatory cytokines and matrix metalloproteinases (MMPs), and decreased neutrophil infiltration. Effective therapeutic management of corneal chemical burns, which progress through distinct phases, requires timely intervention during the early repair phase, where inflammation plays a pivotal role [4].

While various therapeutic techniques have been explored for managing corneal chemical burns, recent research has increasingly focused on mesenchymal stem cells (MSCs) as a promising avenue for the third and fourth phases, thanks to their multipotent nature and diverse range of functions, including tissue repair, immunomodulation, and anti-inflammatory actions [5-7]. This review aims to provide an overview of the evolving strategies and therapies for corneal chemical burns.

## Review

Methodology

**Figure 1:**
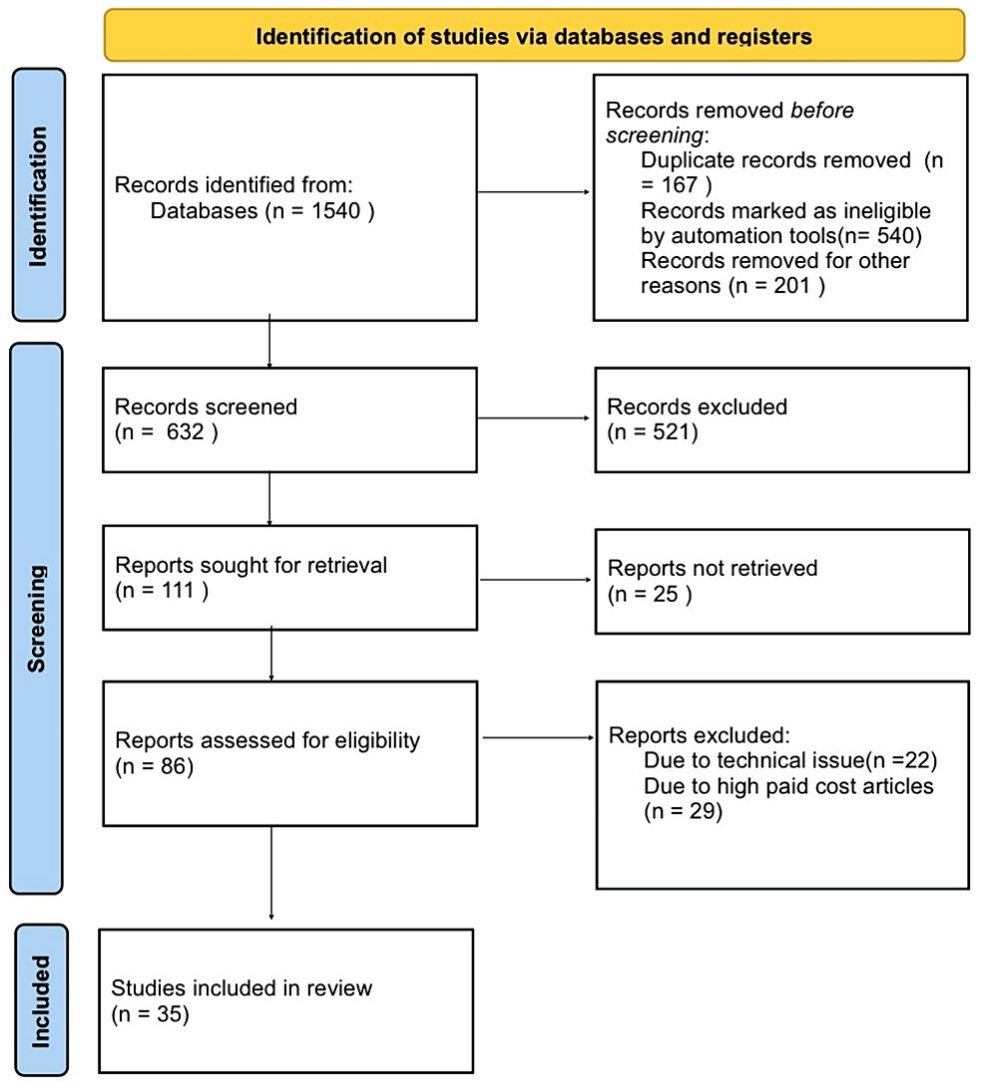
Search strategy utilized for the review

Our research methodology involved a narrative search within the 'PubMed' database, focusing on keywords such as 'cornea,' 'alkali burns,' 'treatment approaches,' and 'nanowafers.' We specifically limited our search to English-language publications. In cases where multiple reports from the same study were identified, we prioritized the most recent findings. Our primary emphasis was on review papers that presented novel discoveries in the field. Figure 1 shows the search strategy followed for the review. 

Discussion

Two studies by Castoro et al., and Chang et al., respectively, aimed to provide an analysis of corneal lymphangiogenesis following alkaline burns, a subject that has lacked substantial documentation thus far. To investigate this, they employed whole-mount corneal imaging, a novel approach that enabled the observation of the processes of corneal lymphangiogenesis and hemoangiogenesis simultaneously, a feature not previously reported. However, it's important to note that this method, while effective, may introduce potential inaccuracies in the quantification of corneal lymphatic vessels. To address this concern, they utilized double-alkaline phosphatase (ALP) enzyme-histochemistry, a more reliable method for distinguishing between blood and lymphatic vessels [8,9].

Chen et al.'s findings revealed a significant correlation between the extent of lymphangiogenesis and hemoangiogenesis in alkali-burned corneas. This suggests that corneas with higher vascularization are more likely to develop lymphatic vessels following alkaline burns. Furthermore, the concurrent emergence of blood and lymph vessels within three days to five weeks post-alkaline burns suggests that assessing corneal hemoangiogenesis can indirectly inform us about the progression of corneal lymphangiogenesis during the post-injury period [10]. However, it's worth noting that the results indicate the presence of corneal blood vessels before the appearance of lymphatics, in contrast to previous models where both blood and lymphatic vessels developed simultaneously.

Alkali burns are known for their adverse effects on corneal epithelial stem cells, leading to corneal vascularization and impaired epithelialization [11]. These consequences are associated with potential complications, such as latent allograft failure. A significant upregulation of vascular endothelial growth factor-C (VEGF-C) expression in corneal tissue following alkali-induced injury was observed. While other factors may contribute, VEGF-C is recognized as the primary driver of lymphangiogenesis. This is supported by previous research on transgenic mice showing increased VEGF-C expression after corneal alkali damage, coinciding with the initiation of corneal lymphangiogenesis [12]. Notably, VEGF-C expression declined after two weeks post-injury, while corneal lymphangiogenesis reached its peak and gradually declined, suggesting a significant role for VEGF-C in the excessive growth of lymphatic vessels in the cornea after alkaline burns. Therefore, targeting VEGF-C with specific interventions may hold promise as a strategy to address this condition [13].

Changes in refractive index upon swelling due to alkali burns

The significant decrease in corneal stromal density caused by tissue swelling has been both theoretically and empirically demonstrated. While there is a clear connection between experimental results and theoretical predictions, it's worth noting that the experimental data displays a less steep slope than the theoretical curve. This phenomenon can be attributed to the limited swelling of the anterior stroma in vitro, resulting in minimal changes to its refractive index during corneal swelling. In contrast, the majority of swelling occurs in the deeper layers beneath the anterior ones [13]. Consequently, alterations in tissue hydration primarily manifest as modifications in the densities of the posterior lamellae. It's important to exercise caution when extrapolating these findings to in vivo corneas, which may exhibit different swelling patterns [14].

An example of bullous keratopathy, characterized by stromal swelling following surgical intervention, is known to coincide with changes in the composition of the extra-fibrillar matrix. Therefore, it's plausible to anticipate that these alterations could potentially affect the refractive index in a manner that deviates from Maumus et al.'s study's projections [14]. However, it's worth noting that the impact of the nonaqueous component in the extra-fibrillar matrix on the refractive index is relatively insignificant due to the high hydration level in the matrix, which further increases as the tissue swells. Maumus et al. developed a straightforward equation linking the refractive index of the corneal stroma to the increase in solvent volume fraction during tissue swelling [14]. It's evident from the information provided that determining the new refractive index requires specifying two parameters: the refractive index of the stroma under physiological hydration conditions and the volume fraction of solvent present in the physiological stroma [15].

On the other hand, when the hydration level becomes exceptionally high, the stromal density must approach that of the solvent. The change in the density of the extra-fibrillar matrix during corneal swelling is expected to impact light scattering [15]. An increase in the refractive index ratio between the fibrils and the matrix may enhance scattering, potentially explaining the increased light beam scattering observed in swollen corneas. Assessing the true extent of this modification is challenging due to concurrent changes in other transmission-dependent characteristics, including fibril packing arrangement and density. Nevertheless, when considering the effects of refractive index alterations alone, assuming that the aforementioned factors remain constant, it's possible to gain insights into the impact of these changes [16].

Treatment modalities

Dexamethasone Nanowafers

Chemical spills often lead to corneal epithelial destruction, opacification, and scarring, resulting in vision loss and potential blindness. In such cases, the primary goal is to reduce ocular inflammation, support corneal epithelium repair, and restore corneal transparency during the acute phase by employing anti-inflammatory medications [17]. Current treatment for eye injuries relies on the application of ophthalmic solutions in the form of topical eye drops. While this method is convenient and noninvasive, it faces challenges due to rapid blinking and tear turnover, leading to the quick clearance of most drugs from the ocular surface, thus limiting treatment effectiveness.

Prior research has shown that both corneal alkali burns and dry eye conditions trigger the release of inflammatory cytokines and elevate MMP activity on the ocular surface. Clinical observations reveal that some patients with ocular burns also experience dry eye syndrome [18-20]. This can be attributed to factors such as impaired conjunctival goblet cells, lacrimal gland dysfunction, reduced tear production, eyelid defects in patients with facial burns, or environmental conditions in ICUs with low humidity and drafts. In Favarin et al. and Ji et al.'s studies, a novel mouse model was established by combining corneal alkali burns and desiccating stress to simulate more severe ocular damage [21,22].

Effective wound healing with Dex-NWs can be challenging due to its ability to inhibit the fibrogenic impact of TGF-β. Dexamethasone, a glucocorticoid with anti-inflammatory properties, is commonly used after corneal damage and ocular surgeries. In outpatient care, the standard treatment for ocular burns involves applying various topical medications, including Dex-NWs, every six hours. One suggested study showed improved visual acuity in corneal alkali burn patients when corticosteroids were administered hourly. The frequency of eye drop administration significantly influences patient compliance; children, elderly patients, and especially those with motor impairments and poor vision exhibit lower levels of adherence. Critically ill patients often rely on caregivers for eye drop administration. Nonetheless, adherence to prescribed treatment protocols plays a crucial role in the prognosis of various ocular conditions [23].

Antioxidant Tualang Honey

Chemical eye injuries often lead to a decrease in the antioxidant defense system and the activation of free radical lipid peroxidation [24]. Surprisingly, there has been limited research on the impact of Asian honey on ocular health. However, a recent study suggests that honey treatment is equally effective as conventional therapy in addressing corneal edema, promoting epithelial healing, and reducing conjunctival hyperemia resulting from alkali chemical injuries [25].

Another promising approach involves dimethylthiourea, an antioxidant topical therapy, which has shown effectiveness in reducing inflammatory responses during acute corneal inflammation [26]. However, neither the honey-treated group nor the conventionally treated group in the study exhibited any clinical signs of infection, including the absence of eye discharge. Despite its high sugar content, honey possesses low water content and acidic properties that inhibit microbial growth. Moreover, when honey is diluted, it generates hydrogen peroxide, contributing to its antibacterial qualities [27].

Interestingly, it has been demonstrated that Tualang honey produces nearly identical results to standard treatment in the context of curing alkali chemical injuries in rabbit eyes. Future research should explore the anti-inflammatory and antioxidant effects with a larger sample size and a control group of rabbits [28].

Cosslinked Thiolated Hyaluronic Acid Film

A study by Niesman et al. assessed the safety, utility, and effectiveness of CMHA-S films as an advanced therapeutic approach for corneal alkali burns. They conducted an evaluation of various parameters, including corneal re-epithelialization, opacity, thickness, and pathology [29]. The research findings affirm the biocompatibility of CMHA-S films. Furthermore, their study demonstrates that the application of CMHA-S treatment significantly enhances corneal re-epithelialization while notably reducing corneal opacity and edema [30]. The process of re-epithelialization in burn injuries is of particular interest due to its clinical implications for ocular burns. During their investigation, untreated burns failed to achieve complete re-epithelialization throughout the trial. In contrast, burns treated with CMHA-S exhibited re-epithelialization within 48 hours. At the 96-hour mark post-injury, both untreated and treated groups showed corneal epithelial abnormalities. Notably, the presence of recurrent erosions of the corneal epithelium is a common observation during corneal alkali burns [31]. However, research indicates that these erosions are not associated with the CMHA-S film treatment.

Prior studies have suggested that hydrogels based on hyaluronic acid (HA) promote re-epithelialization in burn injuries to the extremities. The study by Griffith et al. supports the idea that CMHA-S hydrogel film treatment significantly impacts the re-epithelialization process in corneal burns. It's important to note that other forms of HA, specifically non-crosslinked 0.2% HA drops, do not have a lasting effect on corneal alkali burn healing [32]. However, a drop formulation containing a 1% thiolated crosslinked derivative of HA, known as CMHA-SX, has demonstrated the ability to promote re-epithelialization within 48 hours in cases of corneal alkali burns. Collectively, these findings suggest that treatment outcomes may depend on the specific HA composition, concentration, and mode of delivery [33].

Topically Applied Sodium Hyaluronate

Sodium hyaluronate (Na-HA) is a viscoelastic compound known for its non-inflammatory properties. This molecule is synthesized within cellular membranes and plays various roles in cellular processes, including cell protection, regulation of migration, growth control, cell differentiation, and tissue morphogenesis [34]. It is widely distributed in connective tissues, serving as a significant component of the extracellular matrix. Prior studies have explored the application of topically administered 1% Na-HA in a standardized corneal alkali wound healing model, reporting positive outcomes in the healing process. McCartney and Tschesche objectively assessed the impact of a 1% Na-HA solution on the healing of corneal alkali wounds involving the epithelium, stroma, and endothelium. Their findings reveal the influence of 1% Na-HA application on the infiltration of polymorphonuclear leukocytes (PMNs) and the repopulation of keratocytes during the stromal healing process following alkali burns [35].

## Conclusions

In cases of corneal alkali damage, our review highlighted the occurrence of corneal lymphatic vessel formation, suggesting that transient VEGF-C overexpression in burnt corneas can lead to corneal lymphangiogenesis. The presence of lymphatic vessels in corneas damaged by alkali may exacerbate immunological damage or increase the risk of subsequent transplant rejection. In summary, corneal alkali damage can result from exposure to certain substances, but there are various therapeutic approaches available for treatment. Successful management of corneal swelling and injuries has been achieved through methods such as Dex-NWs, cross-linked hyaluronic acid, antioxidant Tualang honey, and sodium hyaluronic acid. These approaches offer promising options for addressing corneal alkali damage and promoting recovery.
